# Lipoprotein(a) and the Early Diagnosis, Complexity, and Extent of Coronary Artery Disease and Myocardial Infarction

**DOI:** 10.1016/j.jacadv.2025.102542

**Published:** 2026-01-19

**Authors:** Casper F. Coerkamp, Victor A. Verpalen, Kaoutar Bouhbouh, Mick P.L. Renkens, Lars S. Witte, Yannick Kaiser, Remko S. Kuipers, Steven A.J. Chamuleau, Paul Knaapen, Ronak Delewi, Bimmer E.P.M. Claessen, Maik J. Grundeken, R. Nils Planken, Peter Damman, Erik S.G. Stroes, José P.S. Henriques, Nick S. Nurmohamed

**Affiliations:** a Department of Cardiology, Heart Center, Amsterdam University Medical Center, Amsterdam Cardiovascular Sciences, Amsterdam, the Netherlands; b Department of Radiology and Nuclear Medicine, Amsterdam University Medical Center, University of Amsterdam, Amsterdam Cardiovascular Sciences, Amsterdam, the Netherlands; c Department of Vascular Medicine, Amsterdam University Medical Center, Amsterdam, the Netherlands; d Department of Cardiology, Rode Kruis Hospital, Beverwijk, the Netherlands; e Department of Radiology, Mayo Clinic Rochester, Rochester, New York, USA; f Department of Cardiology, Radboud University Medical Center, Nijmegen, the Netherlands

**Keywords:** coronary artery disease, lipoprotein(a), multivessel disease, myocardial infarction, ST-segment elevation myocardial infarction, SYNTAX-1 score

## Abstract

**Background:**

Lipoprotein(a) [Lp(a)] is a potent, independent causal risk factor for coronary artery disease (CAD).

**Objectives:**

This study aimed to assess the association between Lp(a) and the diagnosis, clinical presentation, and angiographic characteristics of obstructive CAD and occurrence of myocardial infarction (MI).

**Methods:**

We included 446 individuals with very high Lp(a) (>230 nmol/L) who underwent routine lipid profiling, matched 2:1 by age and sex using nearest-neighbor propensity matching to 223 controls with low Lp(a) (≤7 nmol/L). Kaplan-Meier analysis was used to assess CAD- and MI-free survival. Multivariable ORs were calculated for multivessel disease and the SYNergy Between percutaneous coronary intervention with TAXus and Cardiac Surgery-1 score.

**Results:**

Median follow-up time, defined by age at last follow-up, was 60 years (Q1-Q3: 50-71). Individuals with very high Lp(a) had significantly lower event-free survival time for the diagnosis of obstructive CAD and occurrence of MI (*P* = 0.006 and *P* = 0.012, respectively). In multivariable analysis, Lp(a) was associated with multivessel CAD (adjusted OR: 1.43 [per 100 nmol/L]; 95% CI: 1.04-1.96; *P* = 0.028), but not with an intermediate or high SYNergy Between percutaneous coronary intervention with TAXus and Cardiac Surgery-1 score (adjusted OR: 1.28 [per 100 nmol/L]; 95% CI: 0.82-1.99, *P* = 0.279). Individuals with very high Lp(a) levels had a 2.4-fold higher risk of ST-segment elevation MI and a 15.9-fold higher risk of recurrent MI compared to those with low Lp(a).

**Conclusions:**

Very high Lp(a) is associated with earlier diagnosis of obstructive CAD and MI, predominantly ST-segment elevation MI. In addition, individuals with very high Lp(a) levels seem at a particular high risk of recurrent MI.

Lipoprotein(a) [Lp(a)], an apolipoprotein B particle covalently linked to apolipoprotein(a), has emerged as a potent, independent, and predominantly genetic risk factor for atherosclerotic cardiovascular disease and aortic valve stenosis.^1^, ^2^, ^3^ Lp(a) concentrations are log-linearly associated with the incidence of myocardial infarction (MI).^4^^,^^5^ Lp(a) concentrations show a linear association with cardiovascular risk; however, a universally accepted cutoff for defining “high” Lp(a) levels has yet to be established.^6^ The atherogenicity of Lp(a) is approximately 5 to 6 times greater than that of low-density lipoprotein cholesterol (LDL-C) particles; a difference that has in part been attributed to its carried load of oxidized phospholipids.^7^

Prior studies have demonstrated that elevated levels of Lp(a) are associated with more severe manifestations of coronary artery disease (CAD), as assessed by invasive coronary angiography (ICA).^8^^,^^9^ Additionally, elevated levels of Lp(a) have also been linked to increased coronary atherogenesis, particularly through the increased development of noncalcified, necrotic-core, thin-cap plaques, which are more prone to rupture and trigger MI.^10^^,^^11^ The effect of Lp(a) on angiographic features and type of MI, including ST-segment elevation MI (STEMI) and non–ST-segment elevation MI (NSTEMI), has not yet been thoroughly investigated.

This study aimed to investigate whether very high Lp(a) levels are independently associated with the early diagnosis, as well as the clinical and angiographic manifestations of CAD and MI subtypes, in a propensity-matched subcohort selected from a tertiary hospital population that underwent clinical lipid profiling.

## Methods

### Study design and population

For this partly retrospective nested subcohort study design, we used a tertiary hospital population as previously described,^12^ in which Lp(a) was routinely measured in all individuals undergoing clinical lipid profiling (n = 12,437) at Amsterdam University Medical Center between October 2018 and October 2019. The present study included 1,000 individuals: 750 with the highest Lp(a) values and 250 randomly selected individuals with low Lp(a) levels from the same sample, thereby enriching the cohort for very high Lp(a) while retaining a representative low-Lp(a) reference group. Patients aged <18 years or with missing clinical data were excluded from the analysis. After selection, the number of eligible individuals with low Lp(a) levels was the limiting factor for matching, allowing a maximum feasible matching ratio of 2:1. Individuals with very high Lp(a) levels were defined by the lowest Lp(a) value remaining within the subset of individuals with the highest Lp(a) values (>230 nmol/L, >93rd percentile) and were matched by age and sex using propensity score matching to individuals with low Lp(a) levels of 7 nmol/L or lower (≤20th percentile; Supplemental Figure 1). The Lp(a) values measured for the purpose of this study were not available to the treating physicians. Decisions to perform ICA were therefore made solely on clinical grounds, minimizing any bias related to knowledge of Lp(a) levels. All data were collected from electronic medical records, with patient consent for data usage obtained via an approved opt-out procedure that was approved by the local ethics committee. The data were updated through December 2024.

### Laboratory measurements

Lipid profiling for all included patients was performed at the local laboratory. Total cholesterol, high-density lipoprotein cholesterol, and triglycerides were measured using enzymatic methods on automated analyzers. LDL-C was measured using the Friedewald formula. Lp(a) levels were measured from the same serum samples as the other lipids using a second-generation assay which is relatively insensitive to kringle-repeats (Roche, Switzerland).

### Angiographic characteristics outcomes

All available ICA images were independently assessed in a random order by 2 investigators (C.C., V.V.) for angiographic evaluation of CAD. In cases of disagreement, a consensus was reached through discussion with an interventional cardiologist with over 20 years of experience as third reader (J.H.). All investigators were blinded to the Lp(a) levels to ensure that the ICA assessments were not influenced. Obstructive CAD was defined as the presence of ≥50% stenosis in at least one coronary vessel with a diameter of ≥1.5 mm on ICA, a history of percutaneous coronary intervention (PCI), or coronary artery bypass grafting (CABG). The SYNergy between PCI with TAXus and Cardiac Surgery (SYNTAX)-1 score was calculated to quantify the anatomical complexity and extent of CAD and was categorized into 3 groups: low (≤22), intermediate (23-32), and high (>32), according to the European Society of Cardiology guidelines.^13^ The extent of CAD also included the number of diseased coronary vessels (classified as single-vessel or multivessel disease) and the number of coronary lesions. Multivessel disease was defined as a stenosis (≥50%) in the left main, or in 2 or 3 coronary vessels. CAD complexity also included the involvement of proximal segments (including left main stenosis) and presence of chronic total occlusion.

### Study outcomes

The coprimary outcomes included time-to-first diagnosis of obstructive CAD in the overall cohort, as well as assessment of CAD complexity and anatomical extent—using the SYNTAX-I score and the presence of multivessel disease—among patients with available ICA images. For the time-to-first diagnosis of obstructive CAD analysis, age was used as the timescale, since Lp(a) levels are generally stable throughout adult life.^14^^,^^15^ The key secondary outcomes were time-to-first event data for the occurrence of fatal or nonfatal MI, using age as the timescale, and the occurrence of recurrent MI, both defined according to the fourth universal definition of MI.^16^ Other secondary outcomes consisted of revascularization with either PCI or CABG, ischemic stroke, and peripheral arterial disease.

### Statistical analysis

Continuous variables are presented as mean ± SD or median (Q1-Q3) depending on the distribution and compared using independent samples *t*-tests. Categorical variables were presented as counts and percentages and compared using the chi-square test. Missing data were imputed by using single imputation methods, as missing values were limited to estimated glomerular filtration rate and cholesterol values (<1% per variable). Given the minimal amount of missing data, formal assessment of missing completely at random assumptions and multiple imputation were not considered necessary. Propensity scores were estimated using logistic regression including age and sex. Nearest-neighbor matching was performed with a 2:1 ratio and a caliper width of 1.0, and balance was assessed for age and sex and descriptively evaluated for other baseline characteristics. For the logistic regression analyses, Lp(a) was analyzed both as a categorical variable using a cutoff value > 230 nmol/L (very high Lp[a] group) and as a continuous variable. Analyses of multivessel disease and the SYNTAX-1 score were performed exclusively among patients with available ICA images, whereas logistic regression analyses for the other outcomes were conducted in the overall cohort. Univariable and multivariable logistic regression analyses were performed to estimate ORs with 95% CIs. The multivariable models were adjusted for age, sex, diabetes mellitus type 2, hypertension, former smoking, total cholesterol, LDL-C, high-density lipoprotein cholesterol, estimated glomerular filtration rate, and lipid-lowering therapy. Age was used as the timescale for all time-to-event analyses. Kaplan-Meier survival curves were used to estimate survival probabilities in the time-to-first event analysis. Time-to-first event was defined as the age at which the first event occurred. Patients were censored at the age of their first event, death, or at the age reached at the time of last follow-up. The log-rank test was applied to compare survival curve distributions between the very high and low Lp(a) groups. A *P* value of <0.05 was considered statistically significant in all analyses. All statistical analyses were conducted using SPSS version 29.0.1 (IBM Corporation) and R version 3.6.0 (R Foundation).

## Results

### Baseline characteristics

A total of 446 individuals with an Lp(a) level of >230 nmol/L were propensity matched to 223 control subjects with ≤7 nmol/L. The mean age of the total population was 56 ± 16 years, and 58.1% were male (Table 1). Baseline characteristics were generally similar between the 2 groups. Patients in the very high Lp(a) group had a higher prevalence of diabetes mellitus type 2 (20.2% vs 13.5%; *P* = 0.033) and a lower prevalence of former smoking (20.2% vs 27.8%; *P* = 0.027) (Table 1).

**Table 1:** Baseline Characteristics Stratified by Lipoprotein(a)

	Total (N = 669)	Lp(a) > 230 nmol/L (n = 446)	Lp(a) ≤7 nmol/L (n = 223)	*P* Value
Age (y) (mean ± SD)	56 ± 16	56 ± 16	55 ± 17	0.566
Male, n (%)	389 (58.1%)	258 (57.8%)	131 (58.7%)	0.825
Diabetes mellitus type 2, n (%)	120 (17.9%)	90 (20.2%)	30 (13.5%)	0.033
Hypertension, n (%)	256 (38.3%)	181 (40.6%)	75 (33.6%)	0.081
Current smoker, n (%)	92 (13.8%)	57 (12.8%)	35 (15.7%)	0.302
Former smoker, n (%)	152 (22.7%)	90 (20.2%)	62 (27.8%)	0.027
HIV infection, n (%)	75 (11.2%)	46 (10.3%)	29 (13.0%)	0.298
Kidney transplant, n (%)	46 (6.9%)	35 (7.8%)	11 (4.9%)	0.160
Lp(a) (nmol/L), median (IQR)	241.3 (7.0-298.3)	273.3 (241.3-332.0)	7.0 (7.0-7.0)	<0.001
Total cholesterol (mg/dL), median (Q1-Q3)	178 (151-213)	182 (151-220)	174 (143-201)	0.004
LDL-C (mg/dL), median (Q1-Q3)	97 (74-128)	101 (74-135)	89 (66-112)	<0.001
HDL-C (mg/dL), mean ± SD	54 ± 19	58 ± 19	54 ± 19	0.257
Triglycerides (mg/dL), median (Q1-Q3)	106 (71-159)	106 (71-159)	115 (71-177)	0.404
eGFR (mL/min/1.73 m^2^), median (Q1-Q3)	75 (59-90)	72 (54-90)	82 (64-90)	<0.001
HbA1c (%), mean ± SD	6.3 ± 1.3	6.3 ± 1.3	6.2 ± 1.3	0.449
BMI (kg/m^2^), mean ± SD	28 ± 15.0	29 ± 19	27 ± 5	0.063
Systolic BP (mm Hg), mean ± SD	135 ± 43	136 ± 51	132 ± 19	0.217
Diastolic BP (mm Hg), mean ± SD	80 ± 12	80 ± 12	79 ± 12	0.120
Lipid-lowering therapy, n (%)	375 (56.0%)	282 (63.2%)	93 (41.7%)	<0.001
Statins, n (%)	341 (51.0%)	259 (58.1%)	82 (36.8%)	<0.001
Ezetimibe, n (%)	121 (18.1%)	102 (22.9%)	19 (8.5%)	<0.001
PCSK9 inhibitors, n (%)	32 (4.8%)	27 (6.1%)	5 (2.2%)	0.029
Fibrates, n (%)	8 (1.2%)	4 (0.9%)	4 (1.8%)	0.314
Antihypertensives, n (%)	302 (45.1%)	198 (44.4%)	104 (46.6%)	0.583
Glucose-lowering therapy, n (%)	128 (19.1%)	93 (20.9%)	35 (15.7%)	0.110
Antiplatelet therapy, n (%)	186 (27.8%)	140 (31.4%)	46 (20.6%)	0.003
Anticoagulants, n (%)	78 (11.7%)	54 (12.1%)	24 (10.8%)	0.609

BMI = body mass index; BP = blood pressure; eGFR = estimated glomerular filtration rate; HbA1c = glycated hemoglobin; HDL-C = high-density lipoprotein cholesterol; LDL-C = low-density lipoprotein cholesterol; Lp(a) = lipoprotein(a); PCSK9 = proprotein convertase subtilisin/kexin type 9.

The median Lp(a) was 273.3 nmol/L (Q1-Q3: 241.3-332.0) in the very high Lp(a) group. The median LDL-C level was higher in the very high Lp(a) group (101 mg/dL [Q1-Q3: 74-135]) compared to the low Lp(a) group (89 mg/dL [Q1-Q3: 66-112]; *P* < 0.008). Total cholesterol levels were higher in the very high Lp(a) group (*P* = 0.004). A greater proportion of patients in the very high Lp(a) group were on lipid-lowering therapy (*P* < 0.001), particularly statins and proprotein convertase subtilisin/kexin type 9 inhibitors, and a greater proportion of very high Lp(a) patients was on antiplatelet therapy (*P* = 0.003) (Table 1).

### Angiographic findings

**Figure 1:**
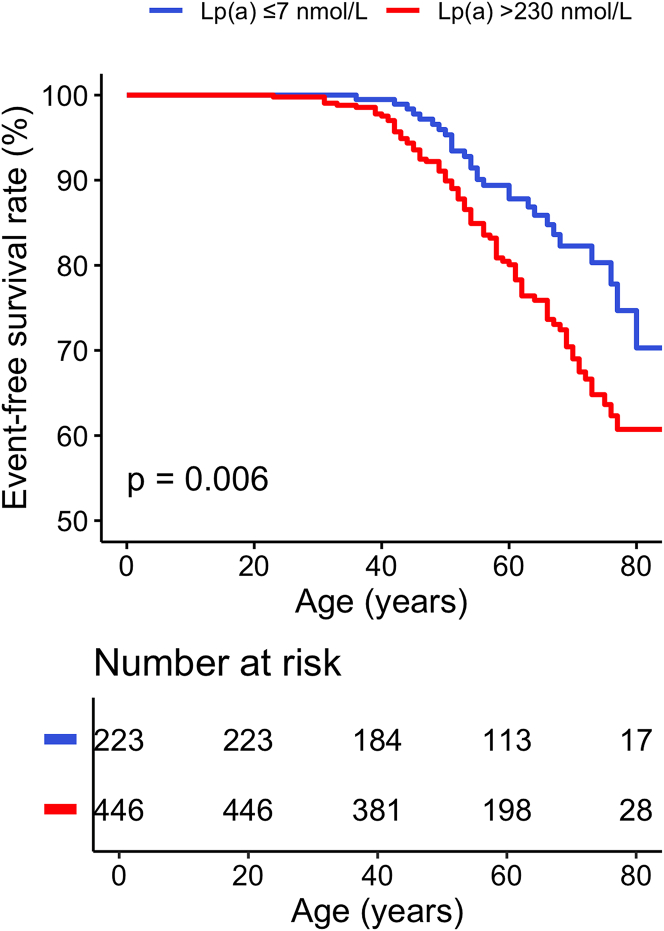
**Diagnosis of Obstructive Coronary Artery Disease According to Lipoprotein(a) Levels** Kaplan-Meier curves according to the very high and low Lp(a) groups for obstructive CAD which was defined as the presence of ≥50% stenosis in at least one coronary vessel with a diameter of ≥1.5 mm on invasive coronary angiography, or a history of percutaneous coronary intervention or coronary artery bypass grafting. CAD = coronary artery disease; Lp(a) = lipoprotein(a).

Using age as the timescale, the very high Lp(a) group demonstrated a higher cumulative incidence of obstructive CAD as assessed by ICA compared with the low Lp(a) group (39.3% [95% CI: 31.1%-46.5%] vs 29.7% [95% CI: 15.6%-41.5%], *P* = 0.006) (Figure 1). The mean age of obstructive CAD detection was 54 ± 12 years in the very high Lp(a) group and 57 ± 11 years in the low Lp(a) group. Among patients with ICA images available (116 [17.3%]), the median number of lesions was higher in the very high Lp(a) group (1 [Q1-Q3: 1-3]) than in the low Lp(a) group (1 [Q1-Q3: 0-2]; *P* < 0.001). Similarly, the median SYNTAX-1 score was higher in the very high Lp(a) group than in the low Lp(a) group (14 [Q1-Q3: 7-24] vs 9 [Q1-Q3: 7-14]; *P* = 0.011). The distribution of low (0-22), intermediate (23-32), and high (>32) SYNTAX-1 scores differed significantly between the 2 groups (*P* = 0.043) (Supplemental Table 1). The multivariable adjusted logistic regression analyses showed that elevated Lp(a) levels were significantly associated with an increased risk of obstructive CAD (adjusted OR: 1.86; 95% CI: 1.08-3.22; *P* = 0.026 and adjusted OR; 1.22 [per 100 nmol/L]; 95% CI: 1.03-1.43; *P* = 0.018) (Table 2). Elevated Lp(a) levels were also associated with an increased risk of multivessel disease in the multivariable analysis (adjusted OR: 1.43 [per 100 nmol/L]; 95% CI: 1.04-1.96; *P* = 0.028, respectively). In the multivariable logistic regression analysis, Lp(a) was not associated with intermediate or high SYNTAX-1 score (Table 2).

**Table 2:** Lipoprotein(a)-Associated ORs for Complexity and Extent of CAD

Analysis	Diagnosis of Obstructive CAD	*P* Value	Multivessel Disease	*P* Value	Intermediate or High SYNTAX-1 Score	*P* Value
Univariate						
Lp(a) >230 nmol/L	1.89 (1.19-2.98)	0.006	2.33 (0.90-6.07)	0.083	2.85 (0.60-13.66)	0.190
Lp(a), per 100 nmol/L	1.19 (1.05-1.35)	0.008	1.34 (1.02-1.76)	0.036	1.24 (0.88-1.75)	0.218
Multivariable model^a^						
Lp(a) >230 nmol/L	2.01 (1.25-3.24)	0.004	2.75 (1.03-7.36)	0.045	3.11 (0.62-15.52)	0.167
Lp(a), per 100 nmol/L	1.24 (1.08-1.43)	0.002	1.44 (1.08-1.92)	0.013	1.31 (0.90-1.89)	0.155
Multivariable model^b^						
Lp(a) >230 nmol/L	1.86 (1.08-3.22)	0.026	2.38 (0.83-6.85)	0.108	1.88 (0.34-10.57)	0.472
Lp(a), per 100 nmol/L	1.22 (1.03-1.43)	0.018	1.43 (1.04-1.96)	0.028	1.28 (0.82-1.99)	0.279

Values are OR (95% CI). Obstructive CAD was defined as the presence of ≥50% stenosis in at least one coronary vessel with a diameter of ≥1.5 mm on invasive coronary angiography, or a history of percutaneous coronary intervention or coronary artery bypass grafting. Multivessel disease is defined as two- or three-vessel disease. Intermediate or high SYNTAX-1 is a score >22.

CAD = coronary artery disease; SYNTAX = the Synergy Between PCI with Taxus and Cardiac Surgery; other abbreviations as in Table 1.

a Adjusted for age and sex.

b Adjusted for age, sex, diabetes, hypertension, former smoking, total cholesterol (continuous), LDL-C (continuous), HDL-C (continuous), eGFR (continuous), and lipid-lowering therapy.

### Clinical outcomes

**Figure 2:**
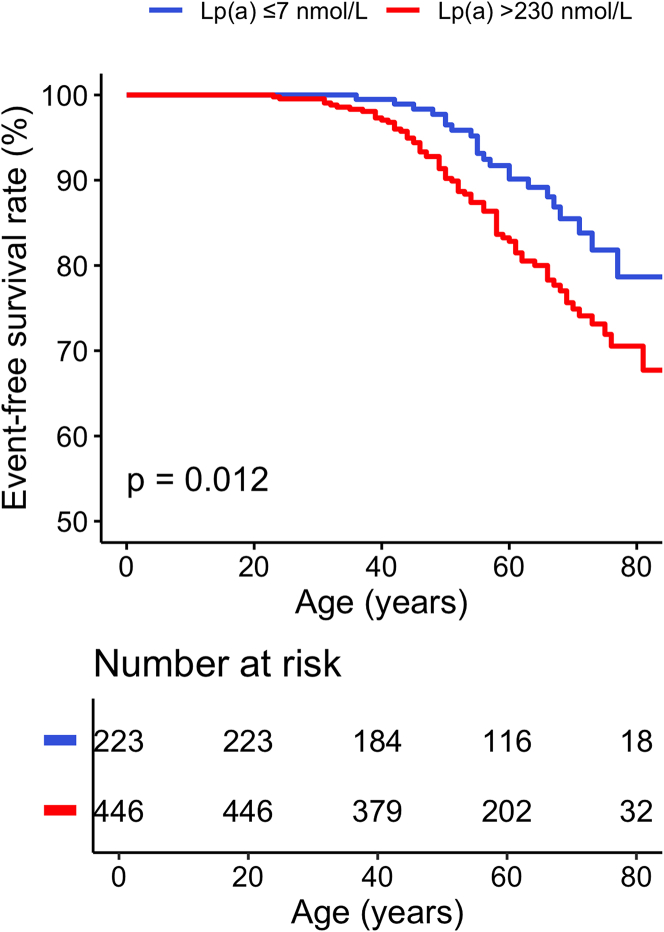
**Incidence of Myocardial Infarction According to Lipoprotein(a) Levels** Kaplan-Meier curves according to the very high and low Lp(a) groups for MI. MI = myocardial infarction; other abbreviation as in Figure 1.

The median age of the total study population at the time of last follow-up was 60 (Q1-Q3: 50-71) years. Using age as the timescale, the very high Lp(a) group demonstrated a higher cumulative incidence of first MI compared with the low Lp(a) group (32.3% [95%: CI: 23.3-40.2] vs 21.3% [95% CI: 11.0-30.5]; *P* = 0.012) (Figure 2). In multivariable analysis, very high Lp(a) was associated with increased odds of first MI (adjusted OR: 1.85; 95% CI: 1.03-3.33; *P* = 0.041) (Table 3). First MI occurring at a younger age in the very high Lp(a) group (mean age 52 ± 12 years vs 57 ± 10 years). The prevalence of STEMI was significantly higher in those with very high Lp(a) levels compared with those with low Lp(a) levels (7.6% [34/446] vs 3.6% [8/223], adjusted OR: 2.42; 95% CI: 1.02-5.72; *P* = 0.044). Conversely, there was no significant difference in the prevalence of NSTEMI between both groups. Particularly, very high Lp(a) levels were strongly associated with recurrent MI, 5.8% (26/446) of patients with very high Lp(a) levels had a recurrent MI, compared to 0.4% (1/223) of patients with low Lp(a) levels (adjusted OR: 15.90; 95% CI: 2.07-122.37; *P* = 0.008). Revascularization procedures, including both PCI and CABG, were more frequently performed in the very high Lp(a) group than in the low Lp(a) group (20.6% [92/446] vs 11.2% [25/223]; *P* = 0.003). Other atherosclerotic cardiovascular disease events, including ischemic stroke and peripheral arterial disease, did not differ significantly between the 2 Lp(a) groups (Table 4).

**Table 3:** Lipoprotein(a)-Associated ORs for Prevalence and Recurrence of Myocardial Infarction

Analysis	Myocardial Infarction	*P* Value	STEMI	*P* Value	Recurrent MI	*P* Value
Univariate						
Lp(a) >230 nmol/L	1.91 (1.15-3.16)	0.012	2.22 (1.01-4.88)	0.047	13.74 (1.85-101.95)	0.010
Lp(a), per 100 nmol/L	1.19 (1.03-1.36)	0.017	1.14 (0.93-1.40)	0.207	1.48 (1.14-1.91)	0.003
Multivariable model^a^						
Lp(a) >230 nmol/L	2.02 (1.20-3.39)	0.008	2.34 (1.05-5.21)	0.037	14.21 (1.91-105.81)	0.010
Lp(a), per 100 nmol/L	1.23 (1.06-1.43)	0.006	1.18 (0.95-1.46)	0.127	1.57 (1.18-2.07)	0.002
Multivariable model^b^						
Lp(a) >230 nmol/L	1.85 (1.03-3.33)	0.041	2.42 (1.02-5.72)	0.044	15.90 (2.07-122.37)	0.008
Lp(a), per 100 nmol/L	1.20 (1.01-1.42)	0.042	1.18 (0.93-1.49)	0.171	1.62 (1.19-2.20)	0.002

Values are OR (95% CI). Myocardial infarction and recurrent MI are defined according to the fourth universal definition of MI. STEMI prevalence was at the time of the first MI.

STEMI = ST-segment elevation myocardial infarction; other abbreviations as in Table 1.

a Adjusted for age and sex.

b Adjusted for age, sex, diabetes, hypertension, former smoking, total cholesterol (continuous), LDL-C (continuous), HDL-C (continuous), eGFR (continuous), and lipid-lowering therapy.

**Table 4:** Clinical Outcomes Stratified by Lipoprotein(a) Group

	Lp(a) >230 nmol/L (n = 446)	Lp(a) ≤7 nmol/L (n = 223)	*P* Value
Diagnosis of obstructive CAD	95 (21.3%)	28 (12.6%)	0.006
Revascularization (PCI or CABG)	92 (20.6%)	25 (11.2%)	0.003
Repeat revascularization (PCI or CABG)	36 (8.1%)	7 (3.1%)	0.014
First myocardial infarction	77 (17.3%)	22 (9.9%)	0.011
STEMI	34 (7.6%)	8 (3.6%)	0.042
NSTEMI	35 (7.8%)	12 (5.4%)	0.239
Silent myocardial infarction	8 (1.8%)	2 (0.9%)	0.367
Recurrent myocardial infarction	26 (5.8%)	1 (0.4%)	<0.001
STEMI	11 (2.5%)	0 (0%)	0.018
NSTEMI	15 (3.4%)	1 (0.4%)	0.020
Ischemic stroke	36 (8.1%)	10 (4.5%)	0.084
Peripheral arterial disease	31 (7.0%)	10 (4.5%)	0.210

Obstructive CAD was defined as the presence of ≥50% stenosis in at least one coronary vessel with a diameter of ≥1.5 mm on invasive coronary angiography, or a history of PCI or CABG. Data are presented as number of patients with percentages in brackets.

CABG = coronary artery bypass grafting; NSTEMI = non–ST-segment elevation myocardial infarction; PCI = percutaneous coronary intervention; other abbreviations as in Tables 2 and 3.

## Discussion

In this unique hospital population of almost 500 individuals with very high Lp(a) levels above the 93rd population percentile, we found that very high Lp(a) levels were associated with earlier diagnosis, greater anatomical complexity as well as greater extent of CAD. Clinically, patients with very high Lp(a) levels had twice as much STEMI compared with patients with low Lp(a) levels, while NSTEMI incidence was comparable. Strikingly, patients with very high Lp(a) levels had 15.9-fold higher risk for recurrent MI compared to those with low Lp(a) levels (Central Illustration). This observation supports existing evidence suggesting that Lp(a) is associated with in-stent restenosis, likely driven by neo-atherosclerosis, and increased plaque vulnerability.^17^^,^^18^ Collectively, these findings shed light on the complex impact of Lp(a) on clinical presentation and outcomes, supporting routine measurement of Lp(a) in individuals presenting to the cardiac catheterization laboratory, especially those patients presenting at younger age and or patients with complex and extensive CAD.

**Central Illustration:**
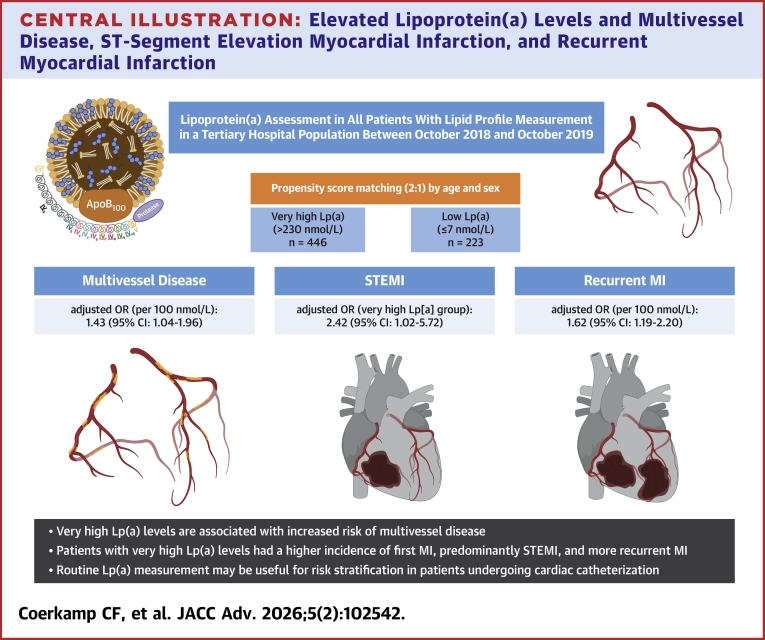
**Elevated Lipoprotein(a) Levels and Multivessel Disease, ST-Segment Elevation Myocardial Infarction, and Recurrent Myocardial Infarction** In the upper panel, a part of the study flowchart is shown. In the lower panel, the risks of multivessel disease, STEMI, and recurrent MI are reported for the very high and low Lp(a) levels. Created in BioRender. STEMI = ST-segment elevation myocardial infarction; other abbreviations as in Figures 1 and 2.

### Lp(a) and complexity and extent of CAD

Utilizing a unique population of patients with very high Lp(a) levels matched to those with low Lp(a) levels, with Lp(a) measurements blinded to the treating clinicians and investigators who assessed the ICA, we demonstrated a persistent relationship between Lp(a) levels and overall CAD burden. These findings indicate that higher Lp(a) concentrations are associated with more extensive and complex atherosclerotic CAD on ICA. This pattern is in line with prior studies showing that elevated Lp(a) more often have more advanced CAD on ICA.^8^^,^^9^ Consistent with those prior studies, our study showed higher SYNTAX-1 scores in patients with very high Lp(a) levels compared to low Lp(a) levels. Conversely, patients with low Lp(a) levels more frequently had less obstructive CAD, and when obstructive CAD was present, they were more often classified as having low SYNTAX-I scores (0-22). In the present study, with Lp(a) measurements blinded to the treating clinicians and investigators who assessed the ICA, patients with very high Lp(a) also had an increased number of coronary lesions compared to low Lp(a). Despite these findings, very high Lp(a) levels were not significantly associated with an intermediate or high SYNTAX-1 score in the multivariable analysis, most likely due to a low number of patients with intermediate-to-high SYNTAX-1 scores (n = 21).

### Pathophysiological association between Lp(a) and increased vulnerable plaque formation

Extent of CAD, plaque vulnerability, or biochemical plaque characteristics, such as inflammation or thrombogenicity, are critical determinants for adverse ischemic outcomes including MI. The Providing Regional Observations to Study Predictors of Events in the Coronary Tree (PROSPECT) II substudy demonstrated that elevated total cholesterol, LDL-C, and non-high-density lipoprotein cholesterol were strongly associated with greater total coronary plaque volume and lipid deposition assessed by intravascular ultrasound and near-infrared spectroscopy, while Lp(a) was uniquely linked to the presence of focal vulnerable plaques, rather than overall plaque burden or lipid deposition. This suggests that Lp(a) promotes a phenotype of CAD characterized by plaque instability and vulnerability.^19^ Our study adds to this evidence by showing a significantly higher incidence of STEMI in patients with elevated Lp(a) levels, in comparison to patients with low Lp(a) levels. This supports the notion that Lp(a) contributes to the formation of unstable plaques that are more prone to rupture, leading to acute MI, particularly STEMI. In contrast, NSTEMI is more commonly attributed to plaque erosion or other mechanisms that lead to myocardial oxygen supply-demand imbalance, rather than to acute plaque rupture.^20^^,^^21^ These pathophysiologic processes may involve mechanisms less directly influenced by Lp(a), but they warrant further investigation in larger studies, as to our knowledge only one other small study with fewer than 100 patients has been performed to date.^22^

### Lp(a) and cardiovascular outcomes

The Kaplan-Meier analysis demonstrated a significantly reduced event-free survival time in individuals with very high Lp(a) levels compared to those with low Lp(a) levels, highlighting the impact of Lp(a) on the early diagnosis of CAD and occurrence of MI. In the multivariable analysis, we demonstrated that very high Lp(a) levels remained a significant predictor of both obstructive CAD and MI. These findings suggest that Lp(a) independently contributes to cardiovascular risk and support its role as a residual risk factor beyond traditional risk factors, consistent with evidence from previous studies.^12^^,^^23^^,^^24^

In the present study, patients with elevated Lp(a) had a 15.9-fold higher risk of recurrent MI, further underscoring its role in ongoing cardiovascular risk. This finding aligns with previous studies reporting a significant association between elevated Lp(a) levels and recurrent ischemic events in patients undergoing PCI.^25^ The combination of Lp(a)’s pro-inflammatory properties and the presence of oxidized phospholipids, which directly drive coronary plaque inflammation, could explain this clinical observation.^26^^,^^27^ This is further supported by growing evidence that Lp(a) contributes to in-stent restenosis and plaque vulnerability, likely mediated by in-stent neo atherosclerosis.^17^^,^^28^ The specific pathophysiological mechanism remains to be fully elucidated; however, Lp(a) binding to the newly formed intima of the artery is thought to promote macrophage infiltration, foam cell formation, and smooth muscle cell proliferation, processes that may contribute to in-stent neo atherosclerosis.^29^^,^^30^

### Clinical implications

Our findings have important implications for clinical risk assessment. The present study shows that individuals with very high Lp(a) levels tend to present with CAD at younger ages in cardiac catheterization laboratories, even in the absence of traditional risk factors.^24^^,^^31^ This underscores the aggressive nature of Lp(a)-mediated atherosclerosis and highlights the importance of proactive management, particularly in light of the cumulative exposure to Lp(a), which can be expressed as “Lp(a)-years.” Despite current guidelines supporting Lp(a) measurement, the lack of specific therapies has resulted in the failure to implement Lp(a) measurement as an integrated part of routine lipid testing in real-world practice.^32^^,^^33^ Our data provide further evidence to consider measuring Lp(a) levels in the cardiac catheterization laboratory, especially in patients with MI, given the high risk of recurrent MI and repeat revascularization. This residual cardiovascular risk for recurrent MI is most pronounced during the first months following the index event; a phase marked by persistent plaque instability and a generalized pro-inflammatory state.^34^ Therefore, routine measurement of Lp(a) in patients with MI may help guide individualized, more intensive secondary prevention strategies.^35^ Although acute-phase variability in Lp(a) appears limited, repeating Lp(a) measurement in the outpatient setting after MI should be considered to establish a true baseline.^22^ Considering that multiple Lp(a)-lowering agents are currently under investigation in phase III clinical outcomes trials, with first results expected in 2026, the identification of individuals with high Lp(a) levels will become even more important; pending the trial outcomes.^36^^,^^37^ Future research should aim to evaluate the effect of Lp(a)-lowering therapies on the MI recurrence risk and in those with in-stent restenosis.

### Study Limitations

This study has several limitations that must be acknowledged. First, this design may introduce survivor bias, as individuals experiencing fatal events prior to cohort inclusion could not be captured. Second, the observational nature of the study precludes conclusions about causality. Additionally, the partly retrospective design, together with the limited availability of ICA at baseline and the absence of follow-up ICA, limits the ability to assess disease progression over time. Third, our study design includes only patients with low and very high Lp(a) levels, which precludes the ability to perform gradient analyses across the full Lp(a) distribution. Fourth, data were obtained from electronic health records, which may have been incomplete or outdated, potentially leading to missing clinical information. However, previous studies have shown that the diagnosis of acute coronary syndromes in electronic health records is generally reliable for research purposes.^38^ Finally, this was a single-center study conducted at an academic tertiary care hospital, which may limit the generalizability of the findings to more diverse patient populations.

## Conclusions

Here, we show that high Lp(a) levels are associated with an earlier diagnosis of obstructive CAD. Additionally, patients with very high Lp(a) levels presented with more complex and extensive CAD, had a higher risk of STEMI, and a strongly elevated risk of recurrent MI. These findings demonstrate the relevance of routine Lp(a) measurement in patients presenting to the cardiac catheterization laboratory as a tool for improved cardiovascular risk assessment and optimal secondary prevention.

Perspectives **COMPETENCY IN MEDICAL KNOWLEDGE:** Patients with very high Lp(a) levels presented with more complex and extensive CAD, had a higher risk of STEMI, and a strongly elevated risk of recurrent MI. These findings support routine measurement of Lp(a) in individuals undergoing cardiac catheterization, particularly younger patients and those with MI, who are at increased risk of recurrent MI and repeat revascularization.

**TRANSLATIONAL OUTLOOK:** Pending the outcomes of Lp(a)-lowering trials, identification of individuals with high Lp(a) levels will become increasingly important. Future studies should aim to evaluate the effect of Lp(a)-lowering therapies on the MI recurrence risk and in those with in-stent restenosis.

## Funding support and author disclosures

Dr Claessen has received speaker fees from 10.13039/100020297Abiomed, and consultancy fees from 10.13039/100002429Amgen, 10.13039/100004339Sanofi, 10.13039/100008497Boston Scientific, and 10.13039/100004320Philips, outside the submitted work. Dr Knaapen has received grants from HeartFlow and Cleerly, outside the submitted work. Dr Stroes has received grants from 10.13039/100002429Amgen, 10.13039/100004336Novartis, and Ionis, and personal fees from 10.13039/100004339Sanofi, 10.13039/100004334Merck, and 10.13039/100004325AstraZeneca, outside the submitted work. Dr Henriques has received research grants from ZonMW, Health ∼ Holland, B. Braun, Infraredx/Nipro, 10.13039/100004325AstraZeneca, and 10.13039/100011949Abbott Vascular, outside the submitted work. Dr Nurmohamed has received grants from the Dutch Heart Foundation (Dekker 03-007-2023-0068) and 10.13039/100016405European Atherosclerosis Society (2023); has received research funding/speaker fees from Cleerly, Daiichi Sankyo, 10.13039/100004336Novartis, and Ultragenyx; and is a co-founder of Lipid Tools. All other authors have reported that they have no relationships relevant to the contents of this paper to disclose.
