# Supplementary material for: Lipoprotein(a) and the Early Diagnosis, Complexity, and Extent of Coronary Artery Disease and Myocardial Infarction
Source: JACC Adv. 2026 Jan 19;5(2):102542. doi: 10.1016/j.jacadv.2025.102542 (PMC12856445; doi:10.1016/j.jacadv.2025.102542)
Supplement: Supplemental Table 1 and Supplemental Figure 1 [file mmc1.docx]

**Supplemental Tables**

**Table 1. Available Angiographic Characteristics by the Lp(a) Groups**

|  | **Lp(a) >230 nmol/L (n = 89)** | **Lp(a) ≤7 nmol/L (n = 27)** | **P-value** |
| --- | --- | --- | --- |
| **Indication ICA** |  |  |  |
| Chronic coronary syndrome | 50 (56) | 19 (70) | 0.263 |
| Acute coronary syndrome ^a^ | 39 (44) | 8 (30) |  |
| **Number of diseased vessels ^b^** |  |  |  |
| No obstructive coronary disease | 20 (22) | 10 (37) | 0.078 |
| Single-vessel disease | 29 (33) | 10 (37) |  |
| Multivessel disease | 40 (45) | 7 (26) |  |
| **Number of lesions (median [Q1-Q3])** | 1 (1-3) | 1 (0-2) | <0.001 |
| **Proximal segment disease** | 48 (54) | 9 (33) | 0.061 |
| **CTO lesion present** | 23 (26) | 6 (22) | 0.704 |
| **SYNTAX-1 score (median [Q1-Q3])** | 14 (7-24) | 9 (7-14) | 0.011 |
| Low (<23) | 50 (72) | 15 (88) | 0.043 ^c^ |
| Intermediate (23-32) | 6 (9) | 2 (12) |  |
| High (>32) | 13 (19) | 0 (0) |  |

Mean ± SD and median (Q1-Q3) are presented depending on distribution. Counts and percentages are used for categorical variables. CTO = chronic total occlusion; ICA = invasive coronary angiography; Lp(a) = lipoprotein(a); NSTEMI = non-ST elevation myocardial infarction; SD = standard deviation; STEMI = ST-elevation myocardial infarction; SYNTAX = the synergy between PCI with taxus and cardiac surgery. ^a^ Acute coronary syndrome was defined as either STEMI, NSTEMI or unstable angina pectoris.
^b^ Every luminal reduction of at least 50% in a coronary vessel with a diameter of ≥1.5mm was defined as a diseased vessel.
^c^ One-way ANOVA was used to compare SYNTAX-1 categories.

**Supplemental Figures**

**Figure 1. Flowchart of Study Design**


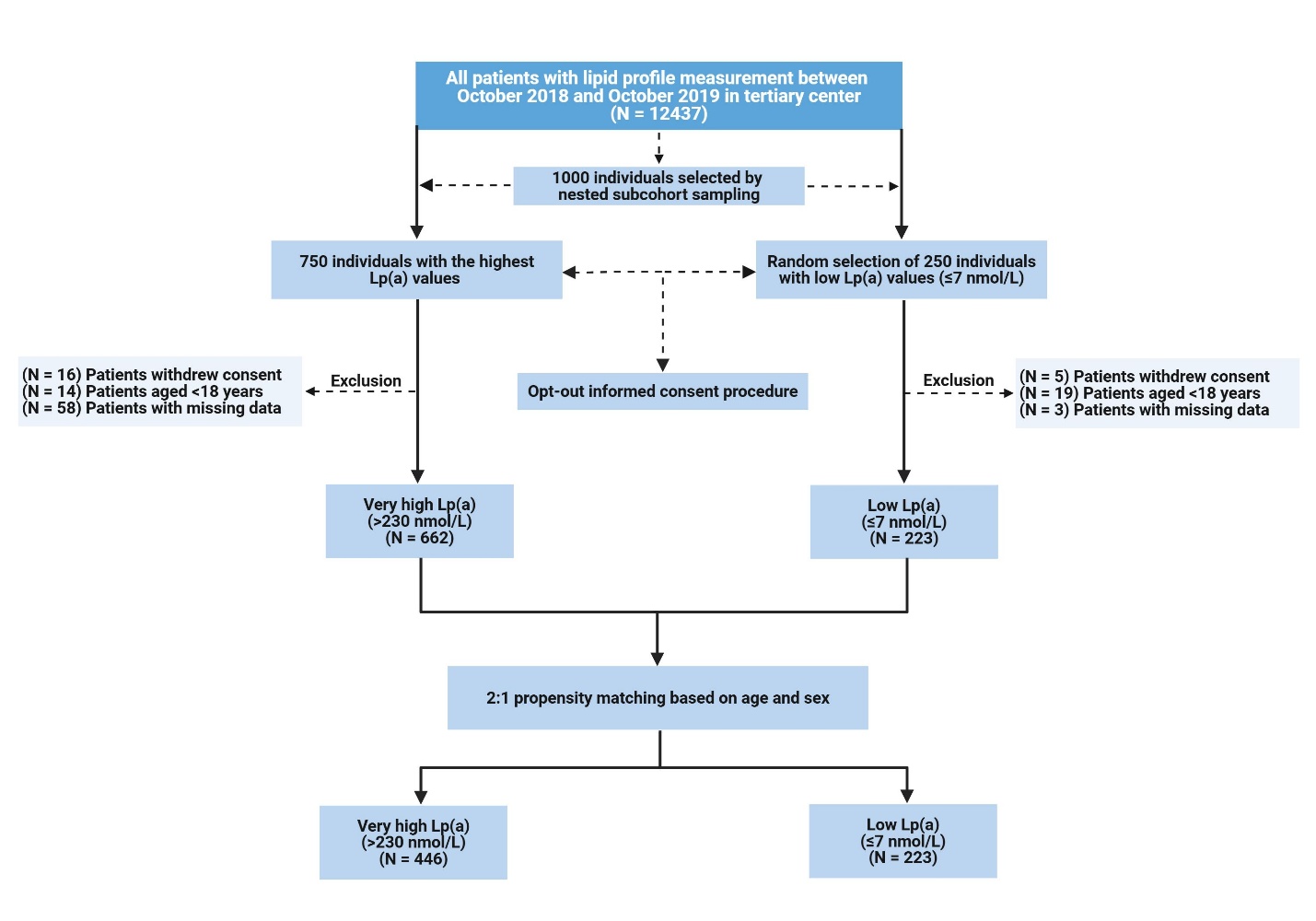


Study flowchart of patients in the very high Lp(a) and low Lp(a) groups, identified through nested subcohort sampling and subsequently matched using propensity scores. Lp(a) = lipoprotein(a).
